# Cadherin signaling: keeping cells in touch

**DOI:** 10.12688/f1000research.6445.1

**Published:** 2015-08-12

**Authors:** Olga Klezovitch, Valeri Vasioukhin

**Affiliations:** 1 Division of Human Biology, Fred Hutchinson Cancer Research Center, Seattle, WA, 98109, USA; 2 Department of Pathology, University of Washington, Seattle, WA, 98195, USA; 3 Institute for Stem Cell and Regenerative Medicine, University of Washington, Seattle, WA, 98195, USA

**Keywords:** cell-cell interactions, cadherin, adherin junctions, cell-cell adhesion, catenin, intracellular signaling pathways

## Abstract

Cadherin-catenin complexes are critical for the assembly of cell-cell adhesion structures known as adherens junctions. In addition to the mechanical linkage of neighboring cells to each other, these cell-cell adhesion protein complexes have recently emerged as important sensors and transmitters of the extracellular cues inside the cell body and into the nucleus. In the past few years, multiple studies have identified a connection between the cadherin-catenin protein complexes and major intracellular signaling pathways. Those studies are the main focus of this review.

## Introduction

The ability of cells to communicate and adhere to each other represents an ultimate prerequisite for the formation and maintenance of a multicellular organism. By sensing their microenvironment, cells can decide whether to continue or stop proliferating, change shape, accept a new identity, move out of the neighborhood, or simply cease to exist. How do the external signals get transmitted inside and prompt the cells to respond accordingly? In the past several years, cadherin-catenin protein complexes emerged as important regulators of morphogenesis and adult tissue homeostasis, linking cell-cell adhesion to multiple major signaling networks. In this short review, we will focus on the most recent studies that address the mechanisms and the functional relevance of the cadherin-mediated intracellular signaling.

## Adherens junctions: structural organization and association with the actin cytoskeleton

**Figure 1.**
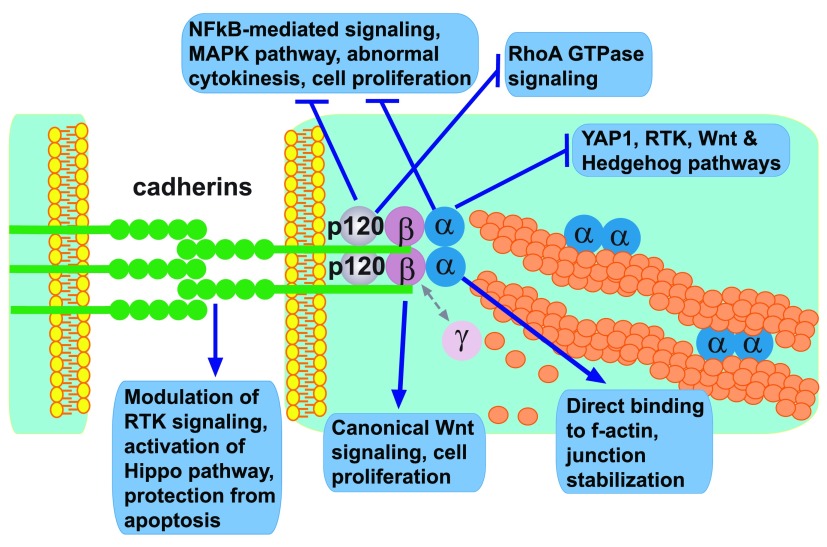
Cadherin-catenin complexes and their role in regulation of major intracellular signaling pathways. The diagram depicts protein members of the adherens junctions clustered at the plasma membranes of two juxtaposed cells and summarizes their individual roles in the intricate network of intracellular signaling pathways. Note that, despite their unique structural features and separate functions, both cadherins and catenins often work in concert and may also participate in the regulation of the same signaling pathway though via a distinct mechanism. Abbreviations: MAPK, mitogen-activated protein kinase; NFκB, nuclear factor-kappa-B; RTK, receptor tyrosine kinase; YAP1, yes-associated protein 1.

Cadherin-catenin complexes comprise the core of a specialized type of adhesion junction named an adherens junction (AJ) ( Figure 1). Among the family of classic cadherins, which includes E (epithelial)-, N (neural)-, P (placental)-, VE (vascular-endothelial)-, R (retinal)-, and K (kidney)-cadherins, E-cadherin is the most frequently employed in the formation of AJs in epithelial cells. To initiate the adhesion process, extracellular domains of cadherins engage in the Ca ^2+^-dependent homophilic trans-interaction with identical cadherin molecules on an adjacent cell, while their cytoplasmic tails bind to p120- and β- (or its homolog γ-) catenin proteins. In turn, β-catenin interacts with α-catenin, which contains an actin-binding domain and physically links AJ complexes to the actin cytoskeleton ^1, 2^. Interaction between the actomyosin cytoskeleton and the AJs is prominently regulated by the mechanical forces and Rho-family of small GTPases (covered in detail in 3– 6). This regulation is necessary for proper tissue morphogenesis and is highly dynamic, facilitating not only the coupling but also the detachment of cadherin-catenin complexes from actomyosin cytoskeleton, allowing cell-cell separation, cell sorting, and cell migration.

## Cadherin-mediated intracellular signaling has a pivotal role in contact inhibition of cell proliferation

The ability of cadherins to transmit signals from the extracellular microenvironment inside the cell body is likely a direct consequence of their adhesive function, which stimulates clustering of cadherin molecules involved in AJ formation. In cell culture experiments, formation of a confluent cell monolayer results in prominent clustering of cadherin-catenin molecules at the AJs. This clustering not only strengthens cell-cell adhesion but also provides important cues for apical-basal cell polarization and significantly influences the downstream signaling events (for review, see 3, 5, 7). It was noticed a long time ago that formation of a confluent cell monolayer results in cell cycle withdrawal ^8^. This phenomenon is known as “contact inhibition of cell proliferation” ^7^. Re-expression of E-cadherin in human epithelial cancer cell lines that lack E-cadherin expression or disruption of E-cadherin with neutralizing antibodies in cell lines that maintained endogenous E-cadherin demonstrated that cadherin-mediated cell-cell adhesion plays a pivotal role in execution of contact inhibition of cell proliferation ^9^. Similarly, activation of cadherin-catenin-mediated cell-cell adhesion by re-expression of α-catenin in a carcinoma line that was missing endogenous α-catenin resulted in retardation of cell proliferation ^10^. A negative impact of E-cadherin expression on tumor progression was also revealed in genetic mouse experiments *in vivo* ^11^. Since restoration of cadherin-catenin-mediated cell-cell adhesion results in prominent changes in cell morphology and re-establishment of apical-basal cell polarity, these early experiments were unable to determine whether cadherin clustering plays a direct or indirect role in negative regulation of cell proliferation. This question was later addressed by elegant experiments in Dr. Gumbiner’s laboratory, which demonstrated that clustering of cellular cadherins by E-cadherin-coated extracellular beads is sufficient to induce proliferation inhibitory signaling, thus directly implicating cadherin clustering in cell signaling events ^12^.

## Cadherin-catenin adhesion and growth factor receptor signaling pathways

How do cadherins exert their signaling functions? Multiple signaling molecules are located at the cell-cell contact sites in direct proximity to the AJ complexes. Many growth- and proliferation-promoting signaling pathways are initiated at the cell surface by receptor-type tyrosine kinases (RTKs). Cadherins can physically interact with several RTKs and they prominently impact their signaling abilities. For example, E-cadherin associates with epidermal growth factor receptor (EGFR) and negatively regulates its kinase activity ^12– 14^. Tumor-suppressor protein neurofibromatosis type 2 (NF2 or Merlin) promotes association between E-cadherin and EGFR, links EGFR to the cortical actin cytoskeleton, and blocks its internalization, which is necessary for EGFR activation and signaling ^15, 16^. Loss of Merlin in mouse liver results in prominent activation of EGFR signaling, expansion of progenitors, and development of liver cancer ^17^. In addition to EGFR, E-cadherin can also negatively impact signaling of other RTKs, including ErbB2, insulin-like growth factor receptor (IGFR), and c-Met ^14^. Similar to E-cadherin in epithelial cells, VE-cadherin in endothelial cells interacts with vascular-endothelial growth factor receptor 2 (VEGFR2) and negatively regulates its mitogen-activated protein kinase (MAPK) signaling by preventing the clathrin-dependent internalization of VEGFR2 and promoting the association between VEGFR2 and tyrosine phosphatase PTPRJ, which dephosphorylates and inactivates VEGFR2 ^18, 19^.

It is important to note that in some cases cadherins can promote growth factor receptor signaling. For example, N-cadherin stimulates fibroblast growth factor receptor signaling by preventing ligand-induced receptor internalization ^20^. Both E-cadherin and VE-cadherin can promote PI3-kinase (PI3K) signaling and protect cells from apoptotic cell death ^21, 22^. VE-cadherin associates with the transforming growth factor-beta (TGF-β) receptor complex and potentiates cell proliferation inhibitory TGF-β signaling events ^23^.

## β- and p120-catenins and the direct line of communication between cell-cell junctions and transcriptional regulation of gene expression

By acting at the plasma membrane, cadherins are ideally positioned to attract and retain their cytoplasmic partners, thus modulating their activation, stability, or nuclear accumulation or a combination of these.

This is important because some of these intracellular proteins are pivotal signaling molecules in their own right. For example, β-catenin is a very potent transcriptional co-activator and a key member of the canonical Wnt signaling pathway (for review, see 24– 26). The levels of cytoplasmic β-catenin available for signaling are tightly controlled by the activity of the β-catenin-destruction protein complex, which is inhibited by activation of Wnt signaling ^24, 25^. Sequestration of β-catenin at the cell junctions can attenuate its ability to enter the cell nucleus and participate in transcriptional regulation. Indeed, multiple studies demonstrated that the loss of cadherin-mediated cell adhesion can promote β-catenin release and signaling ^26^. The exact relationship between cadherin-mediated adhesion and β-catenin signaling is highly complex and context-dependent. In some cases, not only do cadherins not inhibit but they actually potentiate the β-catenin signaling pathway (for review, see 27).

Similarly to β-catenin, cadherins can sequester at the plasma membrane and prevent cytoplasmic accumulation of another member of AJs, p120-catenin (for review, see 28). p120-catenin binds to the transcriptional repressor KAISO and inhibits its function ^29– 31^. In addition, p120-catenin is a potent regulator of Rho-family GTPases and the nuclear factor-kappa-B (NFκB) signaling pathway ^28, 32^. p120-catenin is critical for stabilization of cadherin-catenin complexes and formation of AJs, and this function is likely to be responsible for its tumor-suppressor function in squamous cell carcinoma (SCC), which was revealed by genetic loss-of-function experiments in mice ^33^.

## α-catenin and regulation of cellular signal transduction pathways

α-catenin is crucial for AJ formation because it is necessary for the direct linkage of cadherin-catenin complexes at the membrane with the actin cytoskeleton ^34^. Although there are three α-catenin genes in mammalian genomes (alpha E-catenin *CTNNA1*, alpha N-catenin *CTNNA2*, and alpha T (testis)-catenin *CTNNA3*), most epithelial cells express only one α-catenin ( *CTNNA1*), and the knockout of this gene is usually sufficient for the complete loss of AJ function and loss of cell polarity ^35, 36^. This is different from inactivation of E-cadherin or β-catenin, which may often have redundant functions in the AJs because of the expression of other cadherins and γ-catenin. Notably, this is not the case in the adult heart, where inactivation of all expressed alpha-catenins ( *Ctnna1* and *Ctnna3*) does not cause a severe cell adhesion defect comparatively to N-cadherin knockout mice ^37, 38^.

Similar to p120 catenin ( *Ctnnd1*), genetic loss-of-function experiments in mice revealed prominent tumor-suppressor activity of epithelial α-catenin ( *Ctnna1*), as epidermal stem cell-specific deletion of α-catenin in mice results in the development of SCC tumors ^35, 39, 40^. Like p120-catenin, α-catenin has been linked to NFκB signaling pathway in skin ^39^ and in E-cadherin-negative basal-like breast cancer cells ^41^, where it interacts with and stabilizes IκBα by preventing its ubiquitylation and association with proteasomes ^41^. In addition to its critical role in cell-cell adhesion, via direct interaction with the dynactin protein complex, α-catenin can regulate dynactin-dynein-mediated traffic and integrate the microtubule and actin cytoskeletons during intracellular trafficking events ^42^.

Loss-of-function experiments *in vivo* and *in vitro* revealed an important role of α-catenin in regulation of several major signaling networks, including Ras-MAPK ^35^, canonical Wnt ^27, 43^, and Hedgehog ^44^ pathways. Since α-catenin acts as a tumor suppressor in skin epidermis, our laboratory performed a small interfering RNA (siRNA) screen for genes necessary for this function in keratinocytes, which revealed a connection between α-catenin and yes-associated protein 1 (YAP1), a pivotal target of the Hippo signal transduction pathway ^40^. The connection between cadherin-catenin proteins and the Hippo pathway components has been demonstrated by multiple studies and we will discuss these findings in more detail ^45– 47^ (see below).

## Meet the Hippo: the new darling of the cadherin signaling

First identified in *Drosophila*, the Hippo signaling pathway is evolutionarily conserved and functions as a key regulator of organ size and tumorigenesis by inhibiting cell proliferation and promoting (and, in some cases, inhibiting) apoptotic cell death (for review, see 48, 49). In vertebrates, the core of the canonical Hippo pathway consists of two sequentially acting sets of kinases, MST1/2 and LATS1/2 (Hippo and Warts in *Drosophila*), and several associated co-activators and scaffold proteins. The MST1/2 kinases phosphorylate and activate LATS1/2, which in turn phosphorylates the growth-promoting transcriptional co-activator YAP1 (Yorkie in *Drosophila*) and its homolog TAZ (also known as WWTR1), leading to their cytoplasmic retention. When the Hippo pathway is inhibited, YAP1 translocates to the nucleus, where it binds multiple transcriptional factors and promotes their transcriptional activity ^48, 49^. It is important to note that, in addition to the canonical Hippo signaling pathway, YAP1/TAZ nuclear localization and activity can be regulated independently from MST1/2 and LATS1/2 ^45, 50, 51^. In both *Drosophila* and mammalian model systems, the Hippo signaling is exquisitely sensitive to changes in the actin cytoskeleton or cellular tension which functions as a pivotal regulator that integrates and transmits upstream signals to the Hippo signal transduction pathway (for review, see 49, 52). Increase in F-actin and actomyosin contractility blocks Hippo signaling and prominently activates Yorkie/YAP1/TAZ ^51, 53^.

For a long time, it remained largely unknown whether extracellular cues play any role in activating the Hippo pathway in mammals. The identity of the upstream transmembrane receptors responsible for transmitting the external signals inside the cell was undetermined. Elegant experiments in Dr. Guan’s laboratory identified G-protein-coupled receptors as important upstream regulators of Hippo signaling in mammalian cells ^54^. The evidence that the nuclear localization and activity of YAP1 are inversely correlated with cell density ^55^ pointed in the direction of the cell-cell junctions as potential upstream regulators of the Hippo signaling pathway. Indeed, it was recently demonstrated that E-cadherin homophilic binding at the cell surface in mammalian MDA-MB-231 cells is sufficient to control the subcellular localization of YAP1 independently of other cell interactions ^46^. In addition, two recent studies using primary mouse keratinocytes revealed that α-catenin can bind to YAP1 and sequester it in the cytoplasm, thus modulating the level of YAP1 phosphorylation and its activity ^40, 45^ (for review, see 56, 57). Importantly, there was an inverse correlation between α-catenin levels and nuclear YAP1 localization in both cultured keratinocytes and human SCC tumors, indicating that α-catenin may act as an inhibitor of YAP1 both *in vitro* and *in vivo* ^40^. Of interest, although Ca ^2+^ depletion, which abolishes cadherin homophilic interactions, triggered translocation of YAP1 into the nucleus, the depletion of E/P-cadherin or β-catenin in cultured keratinocytes did not affect the cellular localization of YAP1 ^45^, pointing at the possibility that the expression of other cadherins and catenins might be sufficient to maintain AJs in E/P-cadherin or β-catenin knockdown keratinocytes.

In addition to α-catenin, β-catenin interacts with YAP1 and these proteins prominently impact each other’s nuclear localization and activity ^47, 58, 59^. Constitutive activation of β-catenin in human cancer cells results in the formation of a β-catenin-YAP1-TBX5 transcriptional complex, which is essential for cancer cell survival ^60^.

In *Drosophila*, the Hippo pathway can be regulated by multiple upstream transmembrane modules, which include atypical cadherins Dachsous and Fat (for review, see 61). Recently, another AJ protein, Echinoid, was shown to activate Hippo signaling via its physical interaction with and stabilization of the Hippo-binding partner Salvador ^62^. This interaction is triggered by cell-cell contacts and requires the dimerization of Echinoid cytoplasmic domain. It is of interest to mention that, although there is no known Echinoid homolog in mammals, this protein is able to interact with *Drosophila* E-cadherin, thus contributing to the formation and maintenance of AJs ^63^. Overall, although there are a lot of similarities between *Drosophila* and mammalian Hippo signaling pathways, at least some of the upstream regulators may be quite different ^64^. *Drosophila* Yorkie is missing the C-terminal PDZ-binding motif, which is necessary for the connection between YAP1/TAZ and tight junction (TJ) proteins in mammalian cells. Although α-catenin is a potent negative regulator of YAP1 in mammalian cells ^38, 40, 45, 46, 65^, it is a positive regulator of Yorkie in *Drosophila* ^66, 67^. While E-cadherin is a cell autonomous-positive regulator of Hippo pathway in mammalian cells ^46^, it is a cell autonomous-negative regulator of Hippo in *Drosophila* ^67^. *Fat4*, the mammalian ortholog of *Drosophila fat* gene, does not regulate the Hippo pathway in mouse liver, the organ highly sensitive to changes in the canonical Hippo signaling pathway ^64^. However, mammalian FAT4 and Dachsous cadherins appear to negatively regulate YAP1 in neural progenitor cells ^68, 69^, indicating that at least some of the important connections in Hippo signaling may be tissue- and species-specific.

As discussed above, one of the ways for cadherins to regulate contact inhibition of cell proliferation is by antagonizing the activity of a variety of RTKs, including the EGFR. Interestingly, changes in RTK activity may indirectly impact Hippo signaling. For example, it was recently demonstrated that, in immortalized mammary cells, EGF treatment triggers the nuclear accumulation of YAP1 through activation of PI3K and phosphoinositide-dependent kinase (PDPK1) and this is largely independent of AKT signaling ^70^. Interestingly, in *Drosophila*, EGF signaling also inhibits the Hippo pathway but through a different mechanism, which uses MAPK and the inhibitor of Warts, Jub ^71^. Taken together, those findings point at the important connection between AJs, mitogenic factor pathways, and growth-inhibitory Hippo signaling. Of note, the *Drosophila* Jub was also shown to associate with α-catenin in a cytoskeleton tension-dependent manner, thus linking the actomyosin cytoskeleton, regulation of Hippo pathway activity, and AJs ^66^.

In addition to the AJs, cadherin-mediated adhesion plays an important role in the formation of TJs and the apical-basal cell polarity domains. In turn, the polarity complex proteins can interact with structural components of both AJs and TJs, thus potentially centralizing the regulation of several signaling pathways (for review, see 72), although it is possible that the AJs and cell polarity regulate the Hippo signaling via multiple, genetically separable mechanisms ^67^. The TJ-associated proteins angiomotin and angiomotin-like 1 and 2 directly interact with YAP1/TAZ, localize them to the cytoplasm and TJs, and negatively regulate their transcriptional activity ^73– 76^. Remarkably, at least in some cases, angiomotin proteins promote YAP1 activity by antagonizing YAP1-LATS2 interaction and increasing YAP1 dephosphorylation and translocation to the nucleus ^77^. Interestingly, via its interaction with Merlin, angiomotin can localize to the AJs and facilitate AJ-specific recruitment and activation of LATS ^78^. In both *Drosophila* and mammals, Merlin promotes Hippo signaling by targeting LATS to the cell membrane ^79^. However, since angiomotin proteins are missing in the *Drosophila* genome, the angiomotin-mediated localization and activation of LATS at the AJs are likely to be species-specific, and this may potentially explain the differences in AJ-mediated regulation of YAP1 between *Drosophila* and mammalian model systems.

## Future directions

The unique aspect of cadherin-mediated signaling is that the clustering of cadherin molecules is mediated by the direct cell-cell contacts. This enables cells to identify and map the positions of their immediate neighbors, helping to integrate individual cells into the tissues not only at physical but also at biochemical levels. Although we are continually learning about novel aspects of cadherin-mediated signaling, it is clear that the unifying picture is still not within reach. Knowledge remains highly fragmented with distinct and frequently seemingly opposite findings generated in different model organisms, tissues, or cell culture conditions. Future studies are clearly necessary to accumulate more data in the hope that the sheer quantity of information will inevitably result in a qualitative change in our understanding of how individual cells use their cell-cell adhesion structures to coordinate their behavior in building and homeostatic maintenance of multicellular organisms.

## Abbreviations

AJ, adherens junction; E, epithelial; EGF, epidermal growth factor; EGFR, epidermal growth factor receptor; MAPK, mitogen-activated protein kinase; N, neural; NFκB, nuclear factor-kappa-B; P, placental; PI3K, PI3-kinase; RTK, receptor tyrosine kinase; SCC, squamous cell carcinoma; TGF-β, transforming growth factor-beta; TJ, tight junction; VE, vascular-endothelial; VEGFR2, vascular endothelial growth factor receptor 2; YAP1, yes-associated protein 1.
