# Supplementary material for: Cadherin signaling: keeping cells in touch
Source: F1000Res. 2015 Aug 12;4(F1000 Faculty Rev):550. [Version 1] doi: 10.12688/f1000research.6445.1 (PMC4544379; doi:10.12688/f1000research.6445.1)
Supplement: Peer review history

# Peer review: Referee response for version 1

**DOI:** 10.5256/f1000research.6916.r9953

**Published:** 2015-08-12

**Authors:** Glenn L. Radice

**Affiliations:** 1 Department of Medicine, Thomas Jefferson University, Philadelphia, PA, USA

I have read this submission. I believe that I have an appropriate level of expertise to confirm that it is of an acceptable scientific standard.

# Peer review: Referee response for version 1

**DOI:** 10.5256/f1000research.6916.r9952

**Published:** 2015-08-12

**Authors:** Stefano Piccolo

**Affiliations:** 1 Department of Molecular Medicine, University of Padova, Padova, Italy

I have read this submission. I believe that I have an appropriate level of expertise to confirm that it is of an acceptable scientific standard.

# Peer review: Referee response for version 1

**DOI:** 10.5256/f1000research.6916.r9951

**Published:** 2015-08-12

**Authors:** Andrea I. McClatchey

**Affiliations:** 1 Department of Pathology, Harvard Medical School, Boston, MA, USA

I have read this submission. I believe that I have an appropriate level of expertise to confirm that it is of an acceptable scientific standard.
